# Pleiotropy complicates a trade-off between phage resistance and antibiotic resistance

**DOI:** 10.1073/pnas.1919888117

**Published:** 2020-05-18

**Authors:** Alita R. Burmeister, Abigail Fortier, Carli Roush, Adam J. Lessing, Rose G. Bender, Roxanna Barahman, Raeven Grant, Benjamin K. Chan, Paul E. Turner

**Affiliations:** ^a^Department of Ecology and Evolutionary Biology, Yale University, New Haven, CT 06520;; ^b^BEACON Center for the Study of Evolution in Action, East Lansing, MI 48824;; ^c^Microbiology Program, Yale School of Medicine, New Haven, CT 06520

**Keywords:** bacteriophage, *Escherichia coli*, efflux pump, virus, trade-off

## Abstract

Bacteria frequently encounter selection by both antibiotics and lytic bacteriophages. However, the evolutionary interactions between antibiotics and phages remain unclear, in particular, whether and when phages can drive evolutionary trade-offs with antibiotic resistance. Here, we describe *Escherichia coli* phage U136B, showing it relies on two host factors involved in different antibiotic resistance mechanisms: 1) the efflux pump protein TolC and 2) the structural barrier molecule lipopolysaccharide (LPS). Since TolC and LPS contribute to antibiotic resistance, phage U136B should select for their loss or modification, thereby driving a trade-off between phage resistance and either of the antibiotic resistance mechanisms. To test this hypothesis, we used fluctuation experiments and experimental evolution to obtain phage-resistant mutants. Using these mutants, we compared the accessibility of specific mutations (revealed in the fluctuation experiments) to their actual success during ecological competition and coevolution (revealed in the evolution experiments). Both *tolC* and LPS-related mutants arise readily during fluctuation assays, with *tolC* mutations becoming more common during the evolution experiments. In support of the trade-off hypothesis, phage resistance via *tolC* mutations occurs with a corresponding reduction in antibiotic resistance in many cases. However, contrary to the hypothesis, some phage resistance mutations pleiotropically confer increased antibiotic resistance. We discuss the molecular mechanisms underlying this surprising pleiotropic result, consideration for applied phage biology, and the importance of ecology in evolution of phage resistance. We envision that phages may be useful for the reversal of antibiotic resistance, but such applications will need to account for unexpected pleiotropy and evolutionary context.

## Significance

Bacteriophages (“phages,” viruses that infect bacteria) are an important source of selection for bacterial populations. Phages use various structures to infect bacterial cells, and bacteria often evolve phage resistance by losing or modifying these structures. We examine a phage that uses two structures that also provide *Escherichia coli* cells with antibiotic resistance. We show that phage selection can result in bacteria evolving phage resistance by losing or modifying the structures. When phage resistance evolves, the bacteria sometimes also show increased antibiotic sensitivity. This result indicates an evolutionary trade-off between phage resistance and antibiotic resistance. However, we also discovered bacterial mutations that avoid the trade-off. We discuss the potential use of phage selection and evolutionary trade-offs in treating bacterial infections.

Widespread use of antibiotics in medicine and agriculture has selected for the evolution of multidrug-resistant (MDR) bacterial pathogens (1). Meanwhile, bacteria frequently encounter phages, which are prevalent in the human microbiota, in hospital and farm settings, and in natural environments (2), and which exert selection pressure for bacteria to resist phage exploitation (3–7). However, the interaction between selection from antibiotics and phages, along with its role in driving bacterial evolution, remains unclear, in part because these interactions depend on the environment, phage species, and the bacterial hosts involved in these interactions.

Potential evolutionary interactions between drug resistance and phage resistance mechanisms in bacteria have been previously identified, including both positive and negative interactions that are highly genotype-dependent (8, 9). For example, *Pseudomonas aeruginosa* bacteria that evolve resistance to phage 14/1 simultaneously become more resistant to antibiotics (10), whereas *P. aeruginosa* that evolve resistance to phage OMKO1 become less resistant to antibiotics (5). In *Escherichia coli*, bacteria that evolve resistance to phage TLS also lose antibiotic resistance (11). Such interactions demonstrate that multiple selection pressures sometimes cause bacteria to evolve mutations with trade-up potential (the ability to increase fitness on two traits simultaneously), whereby phages contribute to the problems of increased antibiotic resistance and virulence; in other cases, the mutations have trade-off potential, whereby phages reduce the problem of antibiotic resistance. In both cases of trade-offs and trade-ups, the mutation selected for one function has a pleiotropic effect on another function. This type of pleiotropy, sometimes called *molecular gene pleiotropy*, occurs when a single gene affects multiple traits. For example, the evolution of bacterial resistance to phage can occur via mutations that also decrease cellular resource acquisition (a form of antagonistic pleiotropy) (12), and mutations that improve bacterial growth on one carbon source may improve fitness during growth on another (a form of synergistic pleiotropy) (13). In these ways, pleiotropy can have significant effects on evolution within bacterial populations, shifting phenotypes that are not directly selected.

Pleiotropy in the evolution of phage resistance and antibiotic resistance can result in either decreased (antagonistic pleiotropy) or increased (synergistic pleiotropy) sensitivity to antibiotics. Bacteria−phage interactions can be highly dependent on cell membrane proteins and other surface structures, such as outer membrane proteins (OMPs) and lipopolysaccharides (LPSs), and, in some cases, those structures also contribute to antibiotic resistance. In particular, multidrug efflux pumps are protein complexes spanning the inner and outer membranes of some bacteria, such as the homologous TolC−AcrAB system in *E. coli* and OprM−MexAB system in *P. aeruginosa* (14). These efflux systems confer resistance to multiple antibiotics, acting as generalized transporters for multiple antibiotic classes as well as detergents, dyes, and bile acids (15). The outer membrane protein components (TolC or OprM) are membrane-spanning alpha/beta barrels, with peptide loops that extend outside of the cell. The extracellular loops of OMPs are frequently exploited by phages as the specific binding sites for initiating phage infection (11, 16–18). When phages use these OMPs as receptors, bacteria face selection for reduced or modified OMPs. Phages that use OMPs involved in antibiotic resistance, like the previously characterized TolC-targeting phage TLS (11), might impose selection on bacterial populations to evolve phage resistance while pleiotropically losing antibiotic resistance.

TolC-reliant phages will be useful to the laboratory study of evolutionary trade-offs, or, more practically, may help restore drug sensitivity in clinical settings. To search for such phages, we conducted a screen of our *E. coli* phage library on bacteria that lacked the *tolC* gene, identifying phage U136B. We characterized the molecular interactions between phage U136B and the bacterial outer membrane, identifying that the phage uses two critical host entry factors: TolC and LPS, a structural component of the outer membrane barrier. We then investigated the evolutionary implications of those interactions on the evolution of phage resistance and its pleiotropic effects on antibiotic resistance, comparing phage resistance mutations and phenotypes that initially appear to those that successfully arise during evolution in competitive communities of bacteria and phage.

## Results

### Phage U136B Relies on the Antibiotic Resistance Protein TolC.

**Fig. 1.**
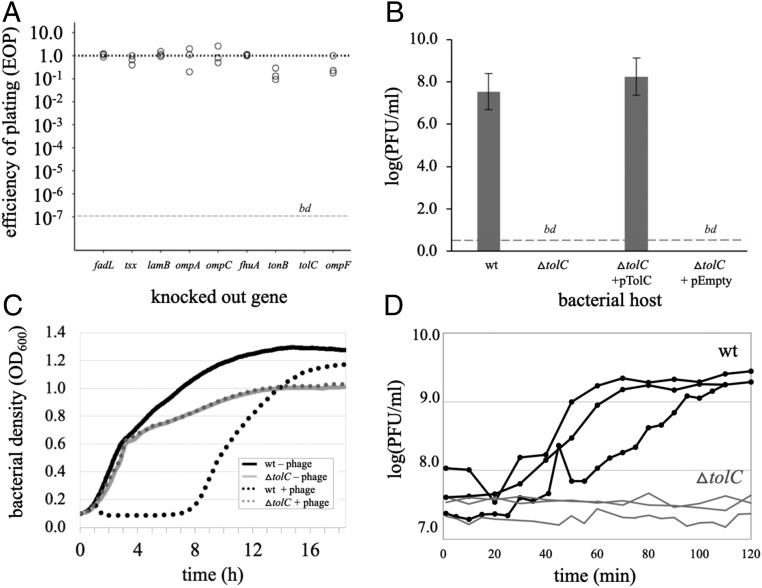
Phage U136B relies on TolC. (*A*) An EOP receptor screen of phage U136B reveals TolC as the candidate OMP receptor. A phage that produces an equal number of plaques on a knockout as on wild-type bacteria has an EOP of 1.0 (dotted line). A “bd” at the lower, dashed line indicates that the EOP was below the limit of detection (∼10^−7^). (*B*) Genetic complementation with a plasmid containing *tolC* fully restores plaquing ability by phage U136B on a *tolC* knockout. Error bars = 95% CIs. (*C*) Bacterial growth curves show that phage U136B lyses wild-type bacteria in liquid culture (∼1.5 h) but has no effect on the *tolC* knockout in liquid culture. (*D*) Single-step growth curves confirm phage U136B cannot grow on a *tolC* knockout in liquid culture.

To identify phages that use the antibiotic efflux pump protein TolC for infection, we screened a collection of *E. coli* phages for the inability to form plaques on a *tolC* knockout. This screen identified phage U136B, a curly-tailed phage with siphophage morphology (*SI Appendix*, Fig. S1) that had been originally isolated from a pig farm in Connecticut (*SI Appendix*, Table S1) and has no plaquing ability on a *tolC* knockout (Fig. 1*A*). Knocking out OMPs used by other phages had no impact on phage U136B infection, indicating *tolC* is unique in this respect. Expression of *tolC* from a plasmid vector fully restores phage U136B plaquing on the *tolC* knockout (Fig. 1*B*). Phage U136B also has no effect on the growth of *tolC* knockout bacteria in liquid culture (Fig. 1*C*), and, correspondingly, the phage cannot grow on the *tolC* knockout (Fig. 1*D*).

### Phage U136B Relies on Inner Core of the LPS.

**Fig. 2.**
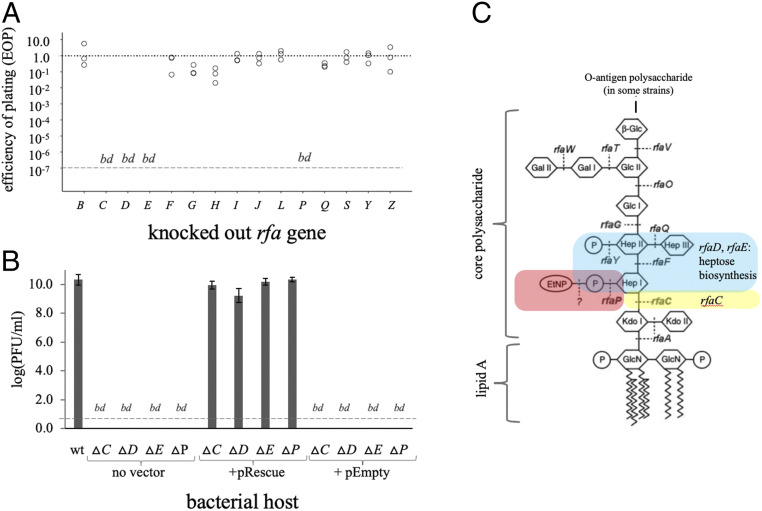
Phage U136B relies on LPS. (*A*) An EOP screen of phage U136B on LPS synthesis gene knockouts reveals genes important to phage replication: *rfaC*, *rfaD*, *rfaE*, and *rfaP*. A “bd” at the lower, dashed line indicates that the EOP was below the limit of detection (∼10^−7^). (*B*) Genetic complementation with plasmids containing respective *rfa* genes fully restores plaquing ability by phage U136B on the knockouts. Error bars = 95% CIs. (*C*) Schematic of genes involved in LPS synthesis, showing that the genes required by U136B affect the deep region of core polysaccharide (regions highlighted in red, blue, and yellow). Modified with permission from refs. 35 and 36, with permission from American Society for Microbiology, with additional data from refs. 37 and 38.

In addition to OMP receptors, many phages also require LPS receptors in their initial contact with the host cell. To assess whether U136B relies on LPS, we conducted a screen on a panel of genes involved in LPS synthesis (the “*rfa*” genes, named originally for the rough [“rf”] colony phenotype of some strains with altered LPS structures). We used all of the *rfa* gene knockouts available in the Keio collection (19) (*SI Appendix*, Table S1), finding that 4 of the 14 *rfa* genes are required by U136B (Fig. 2*A*). We confirmed these genes were required for plaque formation, using genetic complementation tests (Fig. 2*B*). These four genes (*rfa*C, *rfaD*, *rfaE*, and *rfaP*) are all involved in formation of the core region of the LPS polysaccharide component (Fig. 2*C*). Together, these results show that U136B likely requires the inner core of the LPS. Because knocking out either *tolC* alone or any of the *rfa* genes alone provides full protection against U136B, both TolC and LPS appear to be necessary for infection, rather than an “either/or” mechanism.

### Phage Host Range Is Limited to a Subset of *E. coli* Strains.

Phage host range depends largely on receptor availability and receptor structure, so host specificity can be constrained within species. We tested for U136B’s host range on a panel of divergent *E. coli* strains and more-distant Gram-negative bacteria relatives (*SI Appendix*, Table S1). Among the strains we tested for plaquing ability, U136B’s host range was limited to *E. coli* and had strain specificity, with plaquing ability on *E. coli* K, *E. coli* B, and UPEC, but not on a commensal isolate (Table 1 and *SI Appendix*, Table S1) (20).

**Table 1.** Phage U136B has a limited host range within *E. coli*, plaquing on strains with and without the LPS O-antigen

Bacteria	U136B sensitive	*E. coli* O-antigen*	EOP^†^
*E. coli* strains			
BW25113 (K-12 strain, derived from BD792) (39)	+	–	1.0
REL606 (B strain, derived from Bc251) (40)	+	–	3.6 × 10^−1^
CFT073 (UPEC)	+	+	1.78 × 10^−3^
3FM4i (Commensal) (20)	–	+	bd
Other Species			
*Shigella flexneri* PE577	–	NA	bd
*Salmonella enterica* MZ1597	–	NA	bd
*Citrobacter freundii* AB273	–	NA	bd
*P. aeruginosa* PA01	–	NA	bd

EOP is mean of three replicates. NA, not applicable.

* Lists whether any O-antigen is present. *E. coli* O-antigens vary widely among strains and serotypes.

^†^ A listing of “bd” indicates that EOP was below the limit of detection, so these strains are considered entirely resistant to the phage.

### Phage U136B Selects for Altered Cell Surface Factor Genes.

We next looked for *E. coli* phage resistance mutations to determine potential evolutionary paths bacterial populations may take when evolving in the presence of phage. To do this, we used modified fluctuation assays, which reveal preexisting phage resistance mutations that have had minimal time to compete with one another (7). We plated 20 independent cultures of *E. coli* BW25113 (K strain, U136B^S^, Tetracycline^R^, Colistin^R^) with phage U136B, yielding a mutant frequency of 4.0 × 10^−6^. We then randomly picked one phage-resistant mutant colony from each culture. After streaking for double isolation, we sequenced the genomes of each mutant. We observed either *tolC* mutations or LPS-related mutations in 100% of these mutants (Table 2), with LPS-related mutations occurring more frequently (70%, ∼2.8 × 10^−6^) than *tolC* mutations (30%, ∼1.2 × 10^−6^).

**Table 2.** Phage-resistant mutations isolated from the fluctuation experiment

Culture #	Isolate ID	U136B^R^	*tolC* mutation^†^	TET MIC (μg/mL)^‡^	LPS mutation^†^^,^^§^	CST MIC (ng/mL)^¶^	Other genes with mutations^†^^,^^#^
Controls							
BW25113		−		2.0		250	
*tolC* knockout		*+*	*∆tolC732::kan*	0.50		250	
Phage-resistant mutants							
1	RGB-036	+		4.00	*rfaF,* IS1E interruption, coding 162/1,047 nt	100	
2	RGB-040	+	Q158* (CAA→TAA)	0.50		150	
3	RGB-045	+	Δ1 bp, coding 1091/1482 nt	0.50		200	
4	RGB-049	+		4.00	Δ*ghmA* within 48-gene deletion^#^	100	48-gene deletion; see *SI Appendix*, Table S4
5	RGB-058	+		2.00	*rfaG,* IS1E interruption coding 604/1125 nt	100	*ydeM*, C345F (TGC→TTC)
6	RGB-060	+		2.00	*rfaP,* E59* (GAG→TAG)	100	
7	RGB-065	+		4.00	Δ*ghmA* within 48-gene deletion^#^	100	48-gene deletion; see *SI Appendix*, Table S4
8	RGB-071	+	IS5-interupted, coding 190/1482 nt	0.50		200	
9	RGB-074	+		2.00	*rfaP*, IS1E interruption, coding 523/798 nt	150	
10	RGB-079	+		4.00	*rfaD,* Y272N (TAT→AAT)	50	
11	AB350	+	Δ6 bp at 396/1482 nt	0.50		250	
12	AB351	+		4.00	Δ*ghmA* within 48-gene deletion^#^	100	48-gene deletion; see *SI Appendix*, Table S4
13	AB352	+	IS5-interupted, coding 190/1482 nt	0.50		250	
14	AB353	+		4.00	*rfaP*, IS1E insertion, coding 322/798 nt	100	
15	AB354	+		4.00	*rfaP*, Δ1 bp, coding 386/798 nt	100	
16	AB355	+		4.00	*rfaC*, Δ10 bp, coding 582/960 nt	100	
17	AB356	+		4.00	*rfaF*, IS1E interrupted coding 314/1,047 nt	100	
18	AB357	+		4.00	*rfaD,* Δ1 bp, coding 585/933 nt	100	
19	AB358	+	*tolC* IS5 insertion, coding (585/933 nt)	0.50		250	
20	AB359	+		4.00	*rfaF,* IS1E interruption, coding 162/1,047 nt	150	

^†^ A “Δ” indicates deletion of indicated base pairs or gene. An asterisk indicates a point mutation resulting in a stop codon. Values in parentheses indicate either single substitutions or the location of either a stop codon or IS element insertion within coding sequences. Underlined bases indicate single substitutions; nt, indicates nucleotide.

^‡^ Tetracycline (TET) concentrations tested were 0.125, 0.25, 0.5, 1.0, 2.0, and 4.0 μg/mL, so the discrete value listed may be greater than the actual MIC but not more than the next highest value tested. Values are the mode of four to five replicates tested for each mutant.

^**§**^ All *rfa* gene names are synonymous with the corresponding *waa* gene names used in the BW25113 annotation (e.g., *waaP* is equivalent to *rfaP*). Gene *rfaD* is synonymous with the *hldD* gene name used in the BW25113 annotation. We report mutations in the *rfa* form for readability.

^¶^ Colistin (CST) concentrations tested were 12.5, 25, 50, 100, 150, 200, 250, 300, 350, and 400 ng/mL, so the discrete value listed may be greater than the actual MIC but not more than the next highest value tested. Values are the mode of four to seven replicates tested for each mutant.

^#^ The 48-gene deletion included LPS synthesis gene *gmhA* (also called *lpcA*), which codes for a phosphoheptose isomerase involved in LPS synthesis (41–43). A full list of the 48 genes and their annotations is included in *SI Appendix*, Table S4. Gene *ydeM* putatively encodes a YdeN-specific sulfatase-maturating enzyme.

### Phage-Resistant Mutants Have Altered Sensitivity to Antibiotics.

To determine whether the phage-resistant mutants had altered resistance to antibiotics, in particular, decreased antibiotic resistance via antagonistic pleiotropy, we determined their minimum inhibitory concentrations (MICs) for tetracycline and colistin compared to their wild-type, phage-sensitive parental strains. Tetracycline is an antibiotic that binds to the bacterial ribosome and prevents peptide chain elongation. Resistance to tetracycline can occur by efflux through the TolC−AcrAB efflux pump (14), and we predicted that phage resistance mutations in *tolC* would result in decreased resistance to effluxed antibiotics. Colistin is a polypeptide antibiotic in the polymyxin class and is used as a drug of last resort. Previously, a large screen of *E. coli* knockouts suggested that changes to several of the *rfa* LPS synthesis genes may result in increased colistin resistance (21) (summarized in *SI Appendix*, Table S2); here, we predicted that phage resistance mutations in *rfa* genes would result in decreased resistance to colistin.

**Fig. 3.**
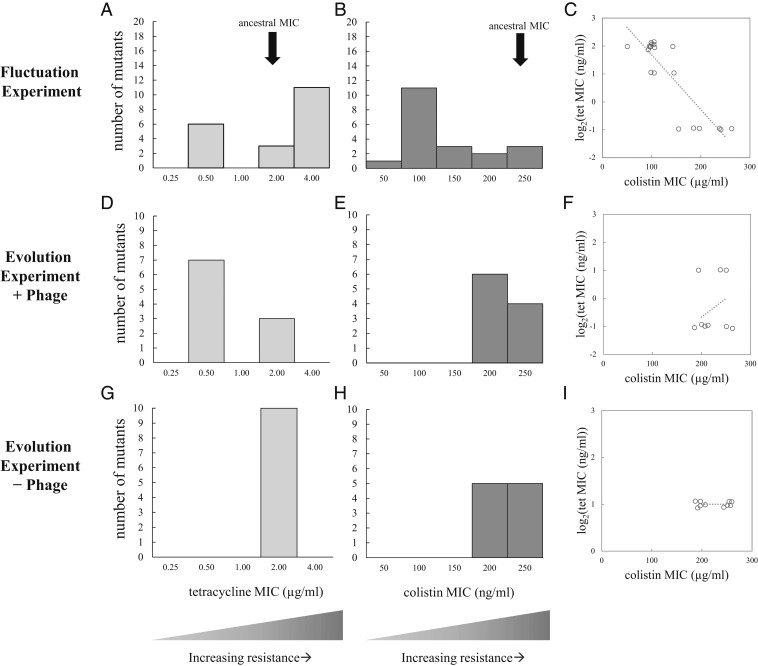
Trade-offs between phage resistance and antibiotic resistance. (*A* and *B*) MICs for phage-resistant isolates from the fluctuation experiment. (*D* and *E*) MICs for phage-resistant isolates evolved in + phage treatment communities. (*G* and *H*) MICs for phage-sensitive isolates evolved in control −phage populations. (*C*, *F*, and *I*) A phage-mediated trade-off between colistin resistance and tetracycline resistance is evident in the fluctuation experiment (in *C*), but this result is alleviated after evolution (in *F*) and doesn’t appear in the control treatment (in *I*). In *C*, *F*, and *I*, a jitter of ±7.5% has been added to the data points for visualization, but regression lines are based on the original, nonjittered data.

Supporting the antagonistic pleiotropy hypothesis, all six *tolC* mutants had reduced resistance to tetracycline compared to the parental strain, and had a similar phenotype to the *tolC* knockout control (Fig. 3*A* and Table 2). Some of the *tolC* mutants also had reduced colistin resistance (Fig. 3*B*, Table 2, and *SI Appendix*, *Supplementary Methods* and Fig. S2). Meanwhile, the other phage-resistant mutants with LPS-related mutations have decreased resistance to colistin (Fig. 3*B*, Table 2, and *SI Appendix*, Fig. S2) compared to the parental strain. Surprisingly, a subset of the LPS mutants had increased resistance to tetracycline (Fig. 3*A* and Table 2) at the same time as their reduced resistance to colistin. This result indicates that the mutant LPS also has a pleiotropic effect on tetracycline resistance (Fig. 3*C* and *SI Appendix*, Table S6).

### Antibiotic Sensitivity Evolves in Populations with Phage Selection.

Our fluctuation experiments revealed bacterial mutations in *tolC* and LPS-synthesis genes, so we next tested whether these same types of mutations would be selected in evolving communities of bacteria and phage. Compared to fluctuation experiments where phage-resistant bacteria have not competed with one another, evolution experiments expose the fitness costs of mutations to selection, altering mutation frequencies over time. The phage resistance mutations may vary in their general fitness costs, in their degree of phage resistance, or in the potential for phages to counteract the various resistance mechanisms via coevolution. We specifically expected that the *tolC* mutations may arise more quickly and outcompete the LPS-synthesis mutants, as growth curve data demonstrated that *tolC* mutants are generally more fit than LPS-synthesis mutants (*SI Appendix*, Fig. S3).

**Fig. 4.**
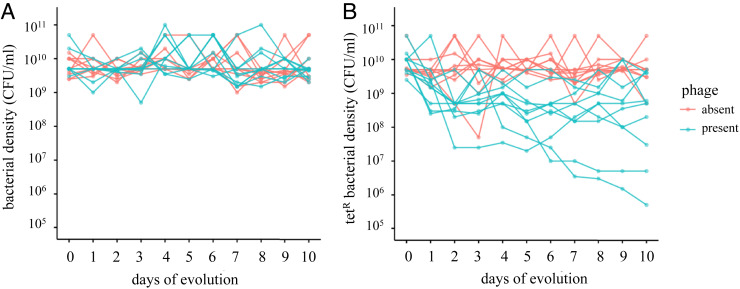
Evolution of antibiotic sensitivity in bacterial populations while under selection for phage resistance. (*A*) Total bacterial population densities and (*B*) tetracycline-resistant bacterial densities in the presence and absence of phage.

To test these ideas, we conducted a 10-d serial passaging evolution experiment. We founded 20 populations with *E. coli* BW25113: 10 populations with phage U136B (“+ phage”) and 10 control populations without phage (“− phage”), each passaged serially for 10 d. Each day, we enumerated total bacterial density (Fig. 4*A*) and tetracycline-resistant bacterial density (Fig. 4*B*) in real time by plating population samples upon serial transfer. During serial passaging, we also monitored phage densities on a daily basis. Phage persisted in all populations through day 5, with half of the phage populations going extinct by day 10 (*SI Appendix*, Table S3).

Again consistent with antagonistic pleiotropy, the populations evolved with phage U136B showed a decreased frequency in the tetracycline-resistant phenotype relative to total population size. This effect was present both before phage extinctions occurred on day 5 [Fig. 4*B*, *t*(10.1) = −6.0, *P* < 0.0001, *t* test of unequal variance] and at the end of the experiment on day 10 [Fig. 4*B*, *t*(11.4) = −3.0, *P* < 0.01, *t* test of unequal variance]. The total bacterial population size was unchanged by phage treatment both before phage extinctions [Fig. 4*A*, *t*(18.0) = 0.82, *P* = 0.789, *t* test] on day 5 and at the end of the experiment [Fig. 4*A*, *t*(11.7) = −1.27, *P* = 0.11, *t* test of unequal variance] on day 10.

To investigate the genetic basis of adaptation and patterns of pleiotropy in the evolved populations, we isolated one random bacterial clone from each of the control (− phage) and treatment (+ phage) populations on day 10. Consistent with our hypothesis that + phage populations would face selection against TolC, after 10 d of evolution, the isolates from the + phage populations showed a predominance of *tolC* mutations and no LPS-related mutations (Table 3). However, we also encountered the unexpected result that 3/10 of these evolved *tolC* mutants also retained the ancestral tetracycline resistance phenotype (Fig. 3*D* and Table 3), indicating these phage resistance mutations had no pleiotropic effect for tetracycline resistance. Those mutations mapped in or near the surface-exposed loops of TolC (22) (*SI Appendix*, Fig. S4), similar to mutations isolated under simultaneous selection by phage TLS and novobiocin (11). We observed few mutations in the − phage control populations, with no mutations in either LPS-related genes or *tolC*. Only isolates evolved in the presence of phage U136B were resistant to the phage (Table 3, U136B^R^), showing that the phage selection was responsible for the evolution of these alleles, and that phage resistance was not a pleiotropic effect of selection in this general environment. The isolates from the control populations also had largely unchanged MICs for tetracycline and colistin (Fig. 3 *G*, *H*, and *I*). To follow up on the tetracycline-resistant population members, we also obtained isolates from cultures plated on tetracycline-containing agar, revealing that the *tolC* mutations do not reach fixation in all + phage populations (*SI Appendix*, *Supplementary Results* and Table S5). Most strikingly, after 10 d of evolution, none of the random + phage population isolates had increased tetracycline resistance, as we had observed for some of the fluctuation isolates (Fig. 3*A* vs. Fig. 3*D*).

**Table 3.** Phage resistance mutations arising during experimental communities with and without phage

Population	Isolate ID	U136B^R^	*tolC* mutation^†^	TET MIC (μg/mL)^‡^	LPS mutation	CST MIC (ng/mL)^§^	Other genes with mutations^¶^
Populations evolved with phage U136B present							
+1	AB277	+	IS5 insertion at 190/1,482 nt	0.25	−	200	*narZ* C292S (TGC→AGC)
+2	AB279	+	Y283D (TAT→GAT)	2.00	−	200	
+3	AB281	+	IS5 insertion coding 190/1,482 nt	0.25	−	200	
+4	AB283	+	21 bp duplication at 259/1,482 nt	2.00	−	250	*ryjB* (novel sRNA, function unknown), T→G at 62/90 nt
+5	AB285	+	IS5 insertion at 194/1,482 nt	0.25	−	200	
+6	AB287	+	G75V (GGC→GTC)	2.00	−	250	
+7	AB289	+	Δ1 bp at 1,066/1,482 nt	0.25	−	200	
+8	AB291	+	E110* (GAA→TAA)	0.25	−	200	
+9	AB293	+	IS5 insertion at 190/1,482 nt	0.25	−	250	*arsC* and *yhiS* Δ1,421 bp at 3,643,975, IS5-mediated
+10	AB295	+	E339* (GAA→TAA)	0.25	−	250	
Populations evolved without phage U136B present							
−1	AB278	−	−	2.00	−	200	
−2	AB280	−	−	2.00	−	200	*bglH* IS*5* (–) +4 bp at 1244/1617 nt
−3	AB282	−	−	2.00	−	250	
−4	AB284	−	−	2.00	−	250	
−5	AB286	−	−	2.00	−	200	
−6	AB288	−	−	2.00	−	200	*metE* L19M (CTG→ATG)
−7	AB290	−	−	2.00	−	200	
−8	AB292	−	−	2.00	−	250	
−9	AB294	−	−	2.00	−	250	*lplT* 24 bp duplication at 229/1194 nt
−10	AB296	−	−	2.00	−	250	

Control MIC values for BW25113 and *tolC* mutant are from the same dataset reported in Table 2.

^†^ A “Δ” indicates deletion of indicated base pairs or gene. An asterisk indicates a point mutation resulting in a stop codon. Values in parentheses indicate either single substitutions or the location of either a stop codon or IS element insertion within coding sequences. Underlined bases indicate single substitutions; nt, indicates nucleotide.

^‡^ Tetracycline (TET) concentrations tested were 0.125, 0.25, 0.5, 1.0, 2.0, and 4.0 μg/mL, so the discrete value listed may be greater than the actual MIC but not more than the next highest value tested. Values are the mode of three to four replicates tested for each strain.

^**§**^ Colistin (CST) concentrations tested were 12.5, 25, 50, 100, 150, 200, 250, 300, 350, and 400 ng/mL, so the discrete value listed may be greater than the actual MIC but not more than the next highest value tested. Values are the mode of two replicates tested for each mutant.

^¶^ *narZ*: nitrate reductase 2 (NRZ), alpha subunit; *ryiB*: novel small RNA (sRNA), function unknown; *arsC*: arsenate reductase; *yhiS*: putative uncharacterized protein; *bglH* is a cryptic carbohydrate-specific OMP gene; *metE* is a 5-methyltetrahydropteroyltriglutamate-homocysteine S-methyltransferase gene; *lplT* is a lysophospholipid transporter gene.

## Discussion

The interactions between bacteriophages, antibiotic-resistant bacteria, and antibiotic-sensitive bacteria are complex. One reason we lack detailed knowledge about their ecological and evolutionary interactions is that few phages have been tested for interactions with antibiotic resistance genes. To gain insight into such relationships, we screened a collection of 33 environmental and commercial *E. coli* phages for their reliance on the antibiotic efflux pump gene *tolC*. The screen identified phage U136B (Table 1), which relies on both the antibiotic resistance gene *tolC* (Fig. 1) and the core of the LPS (Fig. 2). We used fluctuation assays to find that phage U136B selects for host mutations in genes encoding its critical host entry factors, *tolC* and LPS (Table 2). These phage-resistant mutations had pleiotropic changes to their sensitivity to tetracycline (mediated by *tolC* changes), colistin (mediated by LPS-related changes), or both (Table 2 and Fig. 3 *A* and *B*), including both predicted cases of antagonistic pleiotropy and surprising cases of synergistic pleiotropy. Phage resistance mutants and their associated antibiotic sensitivity phenotypes also evolved in communities of *E. coli* and phage U136B (Table 3 and Fig. 3 *D* and *E*).

### The Role of Competition and Community in Pleiotropic Evolution.

Comparing the mutations and phenotypes that arise during fluctuation experiments to those from evolution experiments provides an opportunity to compare the accessibility of specific mutations (in this case, *tolC* and LPS-related mutations) to their actual success during ecological competition and coevolution. Our fluctuation experiments revealed that both tetracycline-sensitive (*tolC*) and colistin-sensitive (LPS-related) mutants arise readily, with LPS-related mutants dominating numerically (Fig. 3 *A* and *B* and Table 2), likely because this complex, multilocus trait has more mutational targets than the single *tolC* gene. In contrast, after 10 d of evolution in a community with phage U136B, LPS-related mutants were relatively rare, appearing in none of the random isolates from the 10 + phage populations. This effect is mediated by fitness differences between *tolC* and LPS-related mutations (*SI Appendix*, Fig. S3). Overall, these results reveal that competition can result in different outcomes than might be expected from a survey of accessible mutations.

The outcomes of competition over evolutionary time will also be influenced by how long phages persist in the treatment environment. While all 10 of our + phage populations started under identical conditions and contained *tolC* mutants, these populations otherwise widely varied in terms of phage persistence (*SI Appendix*, Table S3), the frequency of tetracycline resistance (Fig. 4*B*), and the specific mutations observed (Table 3). Strikingly, the final densities of tetracycline resistance varied by nearly four orders of magnitude across the populations. Together, these results suggest that development of predictable therapeutic phage population dynamics may be complicated, especially since our laboratory conditions will be much less variable than those within and among patients, which would further amplify differences in phage persistence. Furthermore, the dynamics of tetracycline resistance in the + phage populations also varied both before and after phage extinctions, suggesting that the among-population differences were driven in part by the dynamics of competition among the host mutations within the populations (*SI Appendix*, Fig. S3).

Obtaining a better understanding of community evolution and ecological variability will be important for predicting dynamics of therapeutic phages in patients, especially since these complex environments will contain other community members that can impact evolutionary outcomes (23, 24). Such future interdisciplinary work may include incorporating evolutionary factors and environmental heterogeneity into computational models of phage infection, tracking phage population sizes in infection models and clinical trials, and including both pharmacokinetic/pharmacodynamic and phage dosing (rather than relying on a single initial inoculum). Together, these approaches may help to better control phage infection and limit persistence to therapeutic windows.

### The Effects of Specific Phage Resistance Mutations on Pleiotropic Traits.

The specific *tolC* mutations observed varied in their effects on the gene as well as on the resulting tetracycline phenotype. We observed two types of *tolC* phage resistance mutations: one type that lost the efflux function of TolC and another type that retained the efflux function (Table 3 and *SI Appendix*, Table S5). All of the evolved *tolC* alleles that retained efflux function contained either nonsynonymous substitutions or small in-frame indels. These mutants were observed only after a period of evolution, suggesting that TolC function may be under weak selection even in the absence of antibiotic, for example, through TolC’s role in dealing with oxidative stress (14). However, even *tolC* alleles that lost efflux function retained rapid growth rates and high cell densities (*SI Appendix*, Fig. S3), so the importance of nonefflux TolC functions appears to be weak. Future work to apply TolC-specific phages will call for broader knowledge about nonefflux functions of TolC in various environments and the likelihood of bacterial evolution that evades a trade-off between phage resistance and antibiotic resistance.

Our data reveal that phage-resistant mutants with LPS-synthesis gene mutations have decreased resistance to the antibiotic colistin. These data are consistent with the results of a high-throughput screen that identified some *rfa* genes involved in colistin resistance (21) (*SI Appendix*, Table S2). Colistin’s mechanism of action is disruption of the bacterial outer membrane via interactions with LPS (25).

It is possible that phage-selected mutations in *rfa* genes change the LPS structure in such a way that makes it more susceptible to colistin disruption, resulting in the evolutionary trade-off between phage resistance and colistin resistance. Consistent with this result, Hao et al. (26) recently found a similar trade-off in the complementary direction, observing that selection for colistin resistance resulted in reduced phage resistance, indicating that this trade-off may evolve in either direction. Although LPS-related mutations were generally less abundant than *tolC* mutations after experimental evolution with phage U136B, we found that the LPS mutants did not necessarily go extinct (*SI Appendix*, *Supplementary Results* and *SI Appendix*, Table S5), indicating their potential importance in phage treatments of infections.

LPS-related changes may also have unintended, negative consequences in pathogenic bacterial populations. Our mutation screen uncovered phage-resistant mutants that had increased resistance to tetracycline through changes to LPS (Table 2), likely due to a combination of decreased permeability of the outer membrane and reduced expression of OmpF, tetracycline’s uptake porin (27, 28). However, not all LPS-related mutants from the initial screen had increased tetracycline resistance relative to the wild type, suggesting the potential to nudge LPS evolution in a direction amenable to reducing antibiotic resistance. The fitness cost of LPS-related mutations is also more severe than that for the *tolC* mutations (*SI Appendix*, Fig. S3), indicating that a general trade-off between LPS-related phage resistance and bacterial growth may further constrain their evolution.

### The Evolution of Pleiotropic Traits Beyond Antibiotic Resistance.

LPS is a crucial determinant of host range and medically relevant phenotypes like antigenicity and virulence (16, 29). Many phages currently being developed for therapeutic use are reliant on LPS, so understanding the evolutionary implications of phage−LPS interactions will be important to predicting whether and when LPS phage selection may change bacterial virulence and resistance phenotypes. In particular, these interactions are important for predicting how phages will interact with their hosts, including potential collateral damage to commensal bacteria and the evolution of host range expansion during treatment. Additionally, if LPS-related phage resistance also results in reduced LPS-associated virulence, then this resistance mechanism might be a useful means for reducing bacterial load while also evolutionarily reducing infection severity.

Overall, our results highlight the importance of considering community-level effects, such as competition, when measuring pleiotropic effects of phage resistance. We envision that phage U136B and others like it may be useful for the direct treatment of pathogenic infections, but that more work is needed to understand the complexity of pleiotropic interactions among phage host range genes, phage resistance, antibiotic resistance, and other traits in complex environments.

## Methods

### Bacterial Growth Conditions and Media.

We grew bacteria in lysogeny broth (LB) with 10 g tryptone, 5 g yeast extract, and 10 g/L NaCl. LB agar included 15 g/L agar, and LB top agar included 7.5 g/L agar, unless otherwise noted. Overnight culture incubation was performed at 200 rpm shaking at 37 °C.

### Phage Library Screen.

We screened 33 phage isolates collected previously from various sources. We screened each isolate using the plaque spot test on host lawns of wild-type and knockout *E. coli* from the Keio collection obtained from the Yale Coli Genetic Stock Center (*SI Appendix*, Table S1). Those phages for which a difference in plaquing was observed in the library screen were then screened for differences in efficiency of plating (EOP, the number of plaques formed on a mutant divided by the number of plaques formed on BW25113). We determined the EOP for phage U136B on each knockout host relative to BW25113 using top agar that contained 3.8 g/L agar and serial dilution spot tests of 2 μL.

### Mutant Selection Procedure.

To select for phage-resistant bacteria, we mixed wild-type bacteria and phages, then plated them onto LB agar and incubated overnight at 37 °C. To ensure the isolation of independent mutations, each replicate was grown from a single isolated colony. To confirm the multiplicity of infection (MOI, the ratio of phage particles to bacterial cells) on the agar plate, we plated bacteria and phage in triplicate and counted colony-forming unit(s) (cfu) per milliliter and plaque-forming unit(s) (pfu) per milliliter, respectively. From each mutant selection plate, we picked a random colony, plus any colonies of notable morphology, and streaked each onto LB agar plate and incubated at 37 °C overnight. We restreaked the colonies to obtain double-purified isolates and grew each in 10 mL of LB at 37 °C with shaking overnight. We archived freezer stocks of each mutant in 20% glycerol, stored at −80 °C.

### Assessment of Phage Resistance.

We streaked 10 μL of a high titer stock of phage U136B (∼10^9^ pfu/mL) along ∼10-cm lines on LB agar plates. Phage streaks were allowed to dry, and 1-μL samples of overnight cultures of each bacterium were streaked perpendicularly across the phage lines in duplicate. Plates were allowed to dry and incubated overnight at 37 °C. Bacterial streaks were scored for signs of phage lysis. Isolates with signs of lysis were scored as U136B^S^, while isolates without lysis were scored as U136B^R^.

### Bacterial Growth Curves for Phage Resistance Assays.

Cultures of wild type and ∆*tolC* were grown to exponential phase as measured by optical density at 600-nm wavelength (OD_600_) of 0.25 to 0.38. The exponential phase cultures were diluted 1:5 into wells with LB to a total volume of 200 μL with phage to reach the target MOI. Cultures were incubated at 37 °C with shaking at 288 rpm for 18 h, and OD_600_ was read every 2 min using an automated spectrophotometer (Tecan microplate reader).

### Bacterial Growth Curves for Comparison of tolC and LPS-Related Mutants.

Bacterial cultures were preconditioned in LB medium overnight from individual colonies, then transferred to fresh LB in 200-μL total volumes. Cultures were incubated at 37 °C with shaking, and optical density was monitored at 5-min intervals for 24 h by a Tecan microplate reader.

### Single-Step Growth Curves.

Overnight cultures of bacteria were diluted 1:100 into 10 mL of prewarmed LB and subjected to shaking at 200 rpm at 37 °C until exponential phase. These cultures were then inoculated into prewarmed LB at an initial concentration of ∼2 × 10^7^ cfu/mL to begin the assay. Phage were added to an initial concentration of 2 × 10^8^ pfu/mL, for an initial MOI of 10. The assay flasks were incubated with shaking at 200 rpm at 37 °C. Every 10 min, 50-μL samples were removed from each flask, transferred to a Spin-X column (CLS8160 Sigma), and spun at 14,000 rpm for 1 min. At 2 and 12 min, bacterial samples were obtained from flasks with and without phage, and were diluted and plated in duplicate as above, in order to preliminarily assess infection rates. Within a week, each filtered phage sample was diluted in a 1:10 dilution series in sterile LB, and 100-μL diluted samples were plated in soft agar overlay with 4 mL of 7.5 g/L LB soft agar and 100-μL overnight DH5α (*SI Appendix*, Table S1). Plates were incubated at 37 °C, and plaques were counted the following day. For each time step, the dilution step with the greatest number of countable plaques was counted and used for analysis.

### Transmission Electron Microscopy.

Images of high titer phage lysates were collected at the Yale Electron Microscopy facility in the Center for Cellular and Molecular Imaging. Samples were negatively stained with 2% uranyl acetate and imaged with FEG 200-kV transmission electron microscopy.

### Broth Microdilution Method for Colistin MICs.

An original colistin solution (Alfa Aesar) with a concentration of 50 mg/mL was diluted to two times the test concentrations in LB medium. Bacterial cultures were grown from single colonies overnight in 10 mL of LB incubated at 37 °C with shaking. Each culture was diluted to ∼2 × 10^5^ cfu/mL in LB. MICs for colistin were measured by dispensing 100 μL of a given colistin concentration and 100 μL of diluted bacteria into wells of a 96-well plate and incubating at 37 °C with shaking for 18 h. Bacterial growth was scored visually as a binary variable (no growth vs. any visible growth). The lowest colistin concentration at which the well was completely clear was the value recorded as the MIC. For cases where replicates gave different answers, we report the mode.

### Statistical Analysis.

All analyses were performed in R (30) using custom scripts.

### Genome Sequence Analysis.

Genome sequences were collected at the University of Pittsburgh Microbial Genome Sequencing Center. Genomic DNA libraries were prepared using a modified Illumina Nextera library preparation (31). Paired-end reads of 151-base pair (bp) length were collected to a final coverage of ∼100-fold across the reference genome. Sequences were analyzed using breseq with default sequence end-trimming mode (32).

### Complementation Assays for Gene Knockouts.

Electrocompetent *E. coli* cells were made by inoculating 10 mL of LB with 100 μL of overnight bacterial cultures grown in LB. These cells were then incubated and shaken at 37 °C for 2 h to 3 h until they reached an OD_600_ of ∼0.6. Next, the cells were centrifuged at 4,000 rpm for 10 min. The supernatant was poured off, then 10 mL of chilled 10% glycerol was added to the pellet. After resuspending the pellet by vortexing, the cells were centrifuged for 5 min at 4,000 rpm, and then the supernatant was poured off. This glycerol wash cycle was performed four times. Finally, the cells were resuspended in 150 μL of 10% glycerol and were frozen at –80 °C or immediately electroporated. Plasmid DNA from a complete set of *E. coli* K12 ORF archive (ASKA) clones (33) (*SI Appendix*, Table S1) was purified using the QIAprep Spin Miniprep kit from Qiagen (#27104). The electrocompetent cells were then either transformed with an empty plasmid or complemented with a plasmid containing the gene which had been knocked out. These transformations were performed by thawing electrocompetent cells and plasmid DNA and adding 1 μL to 3 μL of DNA (<100 ng of DNA) to the electrocompetent cells. After being chilled, the mixture was pipetted into a chilled cuvette and electroporated. Immediately after electroporation, 500 μL of LB were added to recover the cells. The cells were then incubated for 1 h at 37 °C with shaking. Then 100 μL of the electroporated cells were plated on LB plates containing 30 μg/mL chloramphenicol. These plates were then incubated overnight at 37 °C before isolation of transformed colonies.

### Bacteriophage Enumeration and Efficiency of Plating for Complementation Assays.

Soft agar overlay tubes were prepared by mixing 4 mL of 7.5 g/L soft agar, 4 μL of 30 mg/mL chloramphenicol (for transformed strains only), 20 μL of 100 mM IPTG, and 100 μL of a bacterial overnight culture (grown in LB). These soft agar overlays were poured over LB plates containing 30 μg/mL chloramphenicol. For nontransformed strains, no chloramphenicol was added. A 1:10 dilution series was performed for four phage stocks from 10^−1^ through 10^−7^, and 2 μL of each dilution from each phage stock was spotted onto every bacterial overlay. After these spots were completely dry, the plates were incubated overnight at 37 °C. Finally, pfu per milliliter was calculated for each replicate, and the EOP was calculated by dividing the pfu per milliliter of a test strain by that of the wild type.

### Assessment of Tetracycline Resistance.

Overnight cultures were diluted in 1:10 dilution series in sterile LB, and 2-μL spots of each dilution were immediately plated on LB agar plates containing varying concentrations of antibiotic: 0, 0.12, 0.25, 0.5, 1.0, 2.0, and 4.0 μg/mL tetracycline. Plates were allowed to dry and incubated for 2 d at 37 °C. We recorded the MIC of tetracycline for each isolate as the concentration at which colonies no longer formed at the 10^−5^ spotted dilution. For cases where replicates gave different answers, we report the mode.

### Preparation of Phage Stocks for the Evolution Experiment.

Twenty microliters of cryogenically frozen U136B phage stock was diluted in sterile LB in a 1:10 dilution series. One hundred microliters of each dilution was plated with 100 μL of an overnight culture of DH5α in 4 mL of 7.5 g/L LB soft agar overlay on standard LB agar and incubated overnight at 37 °C. Ten isolated plaques were randomly chosen from the 10^−7^ dilution plate, suspended in 750 μL of sterile LB, and filtered through a 0.22-μm Spin-X column (CLS8160 Sigma) at 14,000 rpm for 1 min. The 10 phage stocks were enumerated by top agar overlay plating and subsequently diluted in sterile LB to a target concentration of 1.0 × 10^7^ pfu/mL. For archiving, 200 μL samples of each replicate phage stock were combined in duplicate with 200 μL sterile 40% glycerol solution and frozen at −80 °C.

### Preparation of Bacterial Cultures for the Evolution Experiment.

Cryogenically frozen *E. coli* strain BW25113 was streaked onto LB agar and incubated overnight at 37 °C. Twenty separate isolated colonies were randomly chosen and suspended in 10 mL of sterile LB in separate flasks. Cultures were incubated overnight at 37 °C, with 200-rpm shaking. Samples of these founding cultures were archived by combining 900 μL of each replicate bacterial culture with 900 μL of sterile 40% glycerol solution in duplicate, and frozen at −80 °C. The experimental populations were established by adding 9.8 mL of sterile LB to 50-mL glass Erlenmeyer flasks and then inoculating with 100 μL of one of the BW25113 bacterial cultures described above (each flask receiving a distinct starting culture) to an estimated concentration of 2 × 10^7^ cfu/mL (assuming final *E. coli* overnight densities in LB are 2 × 10^9^ cfu/mL). For the + phage populations, we then added 100 μL of one of the diluted U136B phage stocks prepared above to 10 of the flasks (each flask receiving a distinct starting culture) to a concentration of 1 × 10^5^ pfu/mL. For the 10 control populations, 100 μL of sterile LB were added in place of phage. Flasks were incubated overnight at 37 °C, with 200-rpm shaking.

### Serial Passaging in the Evolution Experiment.

Each day (24 h ± 2 h), the appearance of each flask was recorded, and 100 μL from each experimental flask were transferred into 9.9 mL of sterile LB in fresh flasks. To reduce risk of contamination, the transfer micropipette was wiped down with 70% ethanol between each transfer, and flasks with phage and without phage were alternated to enable detection of cross-contamination. To archive the evolved samples each day, 900-μL samples of each replicate experimental volume were combined in duplicate with 900 μL of sterile 40% glycerol solution and frozen at −80 °C.

### Daily Enumeration of Bacteria during Evolution Experiment.

Each day, 1:10 serial dilutions of each experimental volume were prepared in sterile 0.85% NaCl up to 10^−8^, and 2-μL spots of each dilution were plated on both standard LB agar plates and LB agar plates prepared with tetracycline. Plates were allowed to dry and then incubated overnight at 37 °C. The following day, the most dilute spot with colonies present was identified, and colonies were counted in order to determine the total bacterial population density and the Tet^R^ bacterial population density for each replicate flask.

### Daily Enumeration of Phage during Evolution Experiment.

The dilution series prepared above was also used to enumerate phage daily. For the 10 flasks with phage, 2-μL spots of each dilution were plated onto 100 μL of an overnight culture of DH5α in 4 mL of 7.5 g/L LB soft agar overlay on standard LB round plates (when square plates were used, 125 μL of DH5α was used in 5 mL of soft agar) and incubated overnight at 37 °C. For the flasks without phage, only the least dilute concentration was plated, as a check for phage contamination. The following day, the most dilute spot with plaques present was identified, and plaques were counted in order to determine the phage titer.

### Isolation of Bacteria from Evolution Experiment.

Two individual isolates were obtained from each population at day 5 and day 10. Isolated colonies were selected in the most dilute spots that contained colonies on the bacterial enumeration plates described above. One colony was picked from the total bacterial population (on the standard LB plates), while one colony was picked from the LB plates containing 0.5 μg/mL tetracycline. The colonies were streaked onto sterile LB plates and incubated overnight at 37 °C. For each isolate, the colony most distant from its neighbors was streaked onto a new sterile LB plate and incubated overnight at 37 °C. For each restreaked isolate, the colony most distant from its neighbors was suspended in sterile LB and incubated overnight at 37 °C, with 200-rpm shaking. To archive the isolated bacteria, 900-μL samples of each bacterial culture were combined in duplicate with 900 μL of sterile 40% glycerol solution and frozen at −80 °C.

### Data Availability.

Raw sequence data have been uploaded to the National Center for Biotechnology Information (NCBI) Sequence Read Archive (SRA) at https://www.ncbi.nlm.nih.gov/sra (accession number PRJNA608759) (34). Other data are available in Datasets S1−S7.

## Supplementary Material

Supplementary File

Supplementary File

Supplementary File

Supplementary File

Supplementary File

Supplementary File

Supplementary File

Supplementary File
