# Supplementary material for: Pleiotropy complicates a trade-off between phage resistance and antibiotic resistance
Source: Proc Natl Acad Sci U S A. 2020 May 18;117(21):11207–16. doi: 10.1073/pnas.1919888117 (PMC7260982; doi:10.1073/pnas.1919888117)
Supplement: Supplementary File [file pnas.1919888117.sapp.pdf]

PNAS
www.pnas.org

Supplementary Information for

Pleiotropy complicates a trade-off between phage resistance and antibiotic resistance

Alita R. Burmeister, Abigail Fortier, Carli Roush, Adam J. Lessing, Rose G. Bender, Roxanna Barahman, Raeven Grant, Benjamin K. Chan, Paul E. Turner

Alita R. Burmeister
Email: alita.burmeister@yale.edu

This PDF file includes:

- Supplementary text
- Figures S1 to S4
- Tables S1 to S6
- Legends for Datasets S1 to S7
- SI References

Other supplementary materials for this manuscript include the following:

- Datasets S1 to S7

www.pnas.org/cgi/doi/10.1073/pnas.1919888117

Supplementary Information Text

Supplementary Results

Analysis of additional evolved isolates. Since tetracycline sensitivity did not fix in the + phage populations within 10 days of evolution, we expected that the tetracycline-resistant portion of the populations would be explained by presence of either ancestral (phage-sensitive) or LPS-related genotypes, as not all changes to LPS result in altered tetracycline resistance. To study the structure of the population fraction that remained tetracycline-resistant (ancestral phenotype), we additionally isolated tetracycline resistant bacteria from each population (Table S5) and assessed them for phage resistance and genome sequence changes.

Among the Tet^R evolved isolates, we observed mutations in both *tolC* and LPS-synthesis gene *rfaP*. In one case, an isolate had no known phage resistance mutation and was phage sensitive, indicating the survival of the sensitive ancestral bacteria alongside the phage resistant bacteria, and providing a means for phage replication and persistence through the 10-day experiment.

Supplementary Methods

Kirby Bauer colistin disk diffusion assay. Mueller Hinton plates were prepared by pipetting 25 mL of Mueller Hinton agar into each plate, then allowed to dry for two days. Plates were then used either immediately or refrigerated until use. Overnight bacterial cultures of each strain were prepared by incubating a single bacterial colony in 10 mL LB broth at 37°C for 18-20 hours. After incubation, 1:10 dilutions of each bacterial strain were prepared by combining 900 µL 0.85% NaCl solution with 100 µL bacterial inoculation. To prepare the disk diffusion plates, 100 µL of each bacterial dilution were pipetted onto a Mueller Hinton plate then spread evenly across the plate with a sterile plastic L spreader. Plates were then allowed to dry and cardboard colistin disks (ThermoFisher, # CT0017B) containing 10 µg colistin were placed in the center of the plates using sterile forceps. Plates were incubated for 20-24 hours at 37°C. Finally, to measure the diameters of the zones of inhibition (ZOI), three straight lines, each at a different angle, were drawn through the center of the ZOI using a ruler. The diameter of the ZOI was measured along the drawn lines.

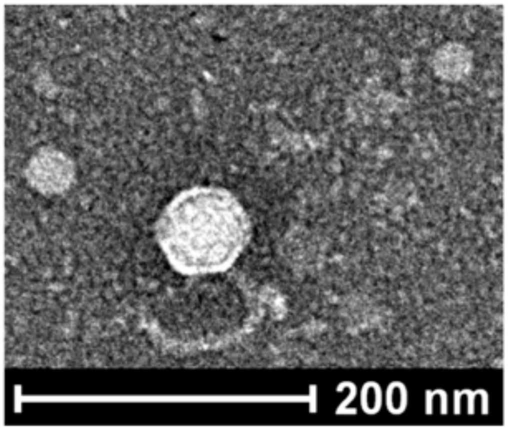

Fig. S1. Transmission electron micrograph showing phage U136B has a siphophage morphology.

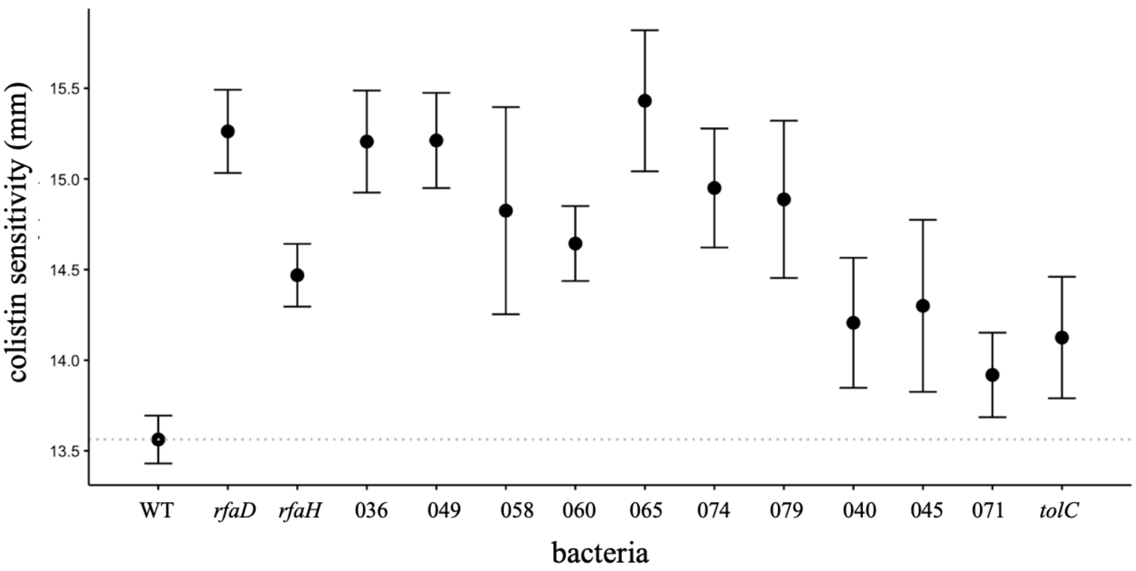

Fig. S2. Kirby-Bauer disk-diffusion assay measurements for colistin sensitivity have high variance but yield similar relative results to the microbroth dilution method. The control strains (Δ*rfaD* and Δ*rfaH*) and phage resistant mutants (RGB-036 to RGB-079) had increased sensitivity to colistin compared to ancestor. Phage resistant mutants RGB-040, 045, and 071 contain *tolC* mutations (main text). Error bars show SEM of 11 to 19 independent measurements of each strain.

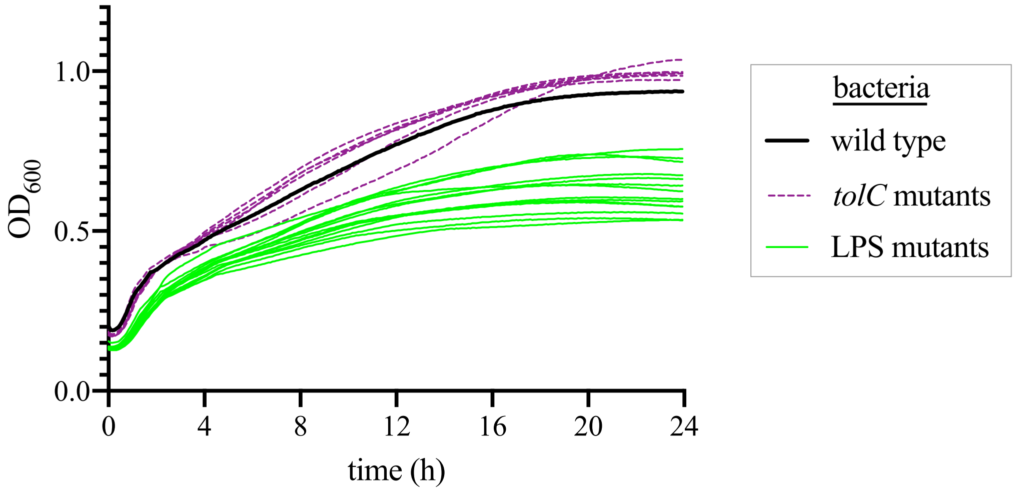

Fig. S3. Phage-resistant mutants vary in fitness costs. Bacterial growth curves include wild type bacteria (BW25113), *tolC* phage-resistant mutants, and LPS-related mutants listed in Table 2 of the main text. Each line shows the mean of three biological replicates.

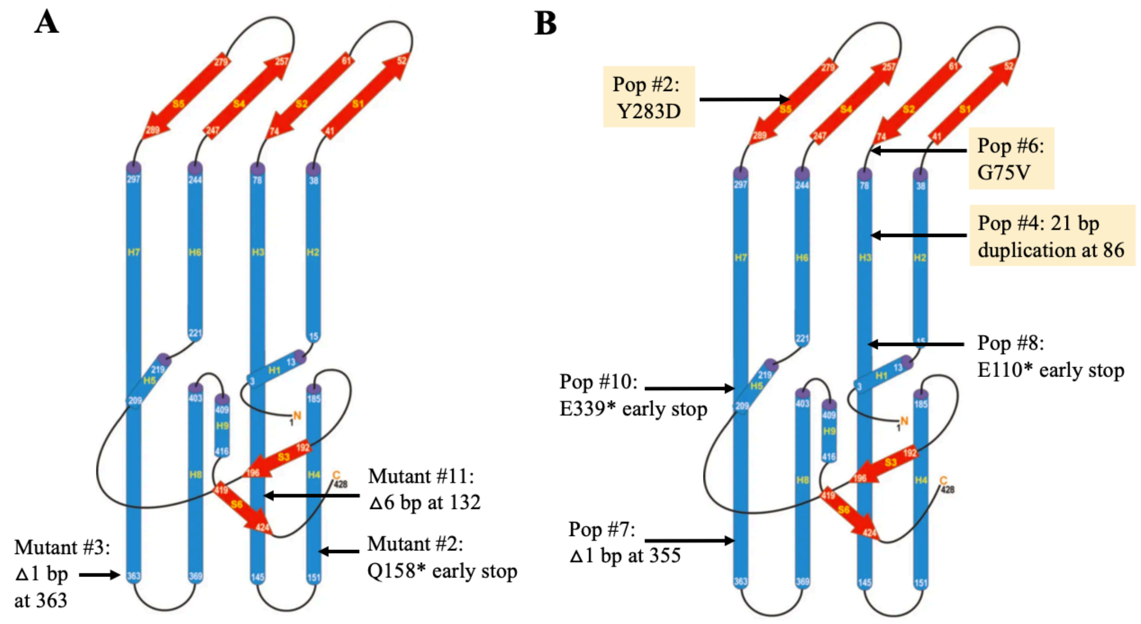

Fig. S4. Location of point mutations and small INDELS in the TolC amino acid sequence for strains from the fluctuation experiment (A) and evolution experiment (B). Mutations highlighted in yellow confer the ancestral tetracycline resistance phenotype (main text Table 3). Insertion-sequence (IS) mediated mutations are note shown. Modified by permission from ref. 1, Springer Nature: *Nature*, copyright (2000).

Table S1. Bacteria and phage used in the study.

Strain	Description	Relevant Characteristics
**Bacteria**		
E. coli		
BW25113	*E. coli* K12, parental strain of Keio collection (19)	
JW5503-1	*tolC* knockout in Keio collection (19)	*ΔtolC732::kan*
JW2341-1	*fadL* knockout in Keio collection (19)	*ΔfadL752::kan*
JW0401-1	*tsx* knockout in Keio collection (19)	*Δtsx773::kan*
JW3996-1	*lamB* knockout in Keio collection (19)	*ΔlamB732::kan*
JW0940-6	*ompA* knockout in Keio collection (19)	*ΔompA772::kan*
JW2203-1	*ompC* knockout in Keio collection (19)	*ΔompC768::kan*
JW0912-1	*ompF* knockout in Keio collection (19)	*ΔompF746::kan*
JW0146-2	*fhuA* knockout in Keio collection (19)	*ΔfhuA766::kan*
JW5195-1	*tonB* knockout in Keio collection (19)	*ΔtonB760::kan*
JW3603-2	*rfaB* knockout in Keio collection (19)	*ΔrfaB739::kan*
JW3596-1	*rfaC* knockout in Keio collection (19)	*ΔrfaC733::kan*
JW3594-1	*rfaD* knockout in Keio collection (19)	*ΔrfaD731::kan*
JW3024-1	*rfaE* knockout in Keio collection (19)	*ΔrfaE745::kan*
JW3595-2	*rfaF* knockout in Keio collection (19)	*ΔrfaF732::kan*
JW3606-1	*rfaG* knockout in Keio collection (19)	*ΔrfaG742::kan*
JW3818-1	*rfaH* knockout in Keio collection (19)	*ΔrfaH783::kan*
JW3602-1	*rfaI* knockout in Keio collection (19)	*ΔrfaI738::kan*
JW3601-3	*rfaJ* knockout in Keio collection (19)	*ΔrfaJ737::kan*
JW3597-1	*rfaL* knockout in Keio collection (19)	*ΔrfaL734::kan*
JW3605-1	*rfaP* knockout in Keio collection (19)	*ΔrfaP741::kan*
JW3607-2	*rfaQ* knockout in Keio collection (19)	*ΔrfaQ743::kan*
JW3604-2	*rfaS* knockout in Keio collection (19)	*ΔrfaS740::kan*
JW3600-1	*rfaY* knockout in Keio collection (19)	*ΔrfaY736::kan*
JW3599-1	*rfaZ* knockout in Keio collection (19)	*ΔrfaZ735::kan*
AG1	ASKA strain collection (33)	Contains pCA24N (“pEmpty”)
JW5503-AM	ASKA strain collection (33)	Contains pCA24N::*tolC*
JW3596-AM	ASKA strain collection (33)	Contains pCA24N::*rfaC*
ASKA(-) rfaC	ASKA strain collection (33)	Contains pCA24N::*rfaD*
JW3024-AM	ASKA strain collection (33)	Contains pCA24N::*rfaE*
JW3605-AM	ASKA strain collection (33)	Contains pCA24N::*rfaP*
REL606	*E. coli* B	
CFT073	Uropathogenic *E. coli* (UPEC), ATCC # 700928	
3FM4i	Commensal strain (20), gift from the lab of Heather Allen-Vercoe (University of Guelph)	
Shigella flexneri PE577	Gift from the lab of Kristin Parent (Michigan State University)	
Salmonella enterica MZ1597		
Citrobacter freundii AB273	AB273, sea turtle isolate	
Pseudomonas aeruginosa PA01		
**Phage**		
Phage U136B	Environmental isolate from swine farm	

Table S2. Sensitivities to colistin in *rfa* gene knockouts. Data summarized from Table S18 of the genome-wide screen reported by Liu et al. 2010 *Antimicrob Agents Chemother* 54(4):1393-1403.

Keio Knockout	Colistin MIC (ng/ml)
Wild type	250
rfaD	100
rfaG	100
rfaC	150
rfaE	160
rfaH	175

Table S3. Phage extinction dynamics during the evolution experiment. Timing of phage extinctions was based on plating filtered phage samples as spot tests, top agar overlays, or both. At Day 10, Population +9 was not detectable in the serial dilution spot test, but it did have plaques appear in the filtered phage sample.

Day	Populations Extinct
1-5	None
6	+1, +3, +5
7	+1, +3, +5, +7, +10
8	+1, +3, +5, +7, +10
9	+1, +3, +5, +7, +10
10	+1, +3, +5, +7, +10

Table S4. Genes in the 48-gene deletion. The only gene readily related to LPS synthesis is *gmhA* (row 6), a phosphoheptose isomerase involved in core polysaccharide synthesis.

Gene	Protein Annotation (Uniprot)
yafU	Putative inner membrane protein
yafF	Putative uncharacterized protein
yafV	Omega-amidase
ivy	Inhibitor of vertebrate lysozyme
fadE	Acyl-coenzyme A dehydrogenase
gmhA	Phosphoheptose isomerase
yafJ	Putative glutamine amidotransferase
yafK	Putative L, D-transpeptidase
yafQ	mRNA interferase toxin
dinJ	Antitoxin
yafL	Probable endopeptidase
rayT	REP-associated tyrosine transposase
lfhA	Putative truncated flagellar export/assembly protein
lafU	Putative truncated flagellar export/assembly protein
dinB	DNA polymerase IV
yafN	Antitoxin
yafO	mRNA interferase toxin
yafP	Uncharacterized N-acetyltransferase
ykfJ	Putative uncharacterized protein
prfH	Putative peptide chain release factor homolog
pepD	Cytosol non-specific dipeptidase
gpt	Xanthine phosphoribosyltransferase
frsA	Esterase
crl	Sigma factor-binding protein
phoE	Outer membrane porin
proB	Glutamate 5-kinase
proA	Gamma-glutamyl phosphate reductase
thrW	Unknown
ykfN	Unknown
ykfI	Toxin
yafW	Antitoxin
ykfH	Uncharacterized protein
ykfG	Unknown
yafX	Uncharacterized
ykfF	Unknown
ykfB	L-Ala-D/L-Glu epimerase (in *Bacillus subtilis)*
yafY	Lipoprotein
ykfL	Uncharacterized
ykfK	Unknown
yafZ	Unknown
ykfA	Uncharacterized
perR	HTH-type transcriptional regulator
insN	Putative transposase for insertion sequence element IS911A
insI1	Transposase for insertion sequence element IS30A
insN	Transposase for inserstion sequence element IS911 (in *Shigella dysneteriae*)
eyeA	Unknown
ykfC	Unknown
insH1	Transposase for insertion sequence element IS5A

Table S5. Mutations in experimentally evolved Tet^R isolates.

Isolate ID	Population	*tolC* mutation[a]	LPS-related mutation[b]	Other genes with mutations[a]
AB297	+1	Δ21 bp, coding (239-259/1482 nt)		
AB299	+2	Δ6 bp, coding (220-225/1482 nt)		
AB301	+3		*rfaP* IS*5* (+) +4 bp, coding (359-362/798 nt)	
AB303	+4	(ACGGCATCAACTCTAACGCGA)$_{1\to2}$ coding (259/1482 nt)		*gatR*[c] insE1 insertion pseudogene (173/475 nt)
AB305	+5	G302D (GGC→GAC)		
AB307	+6	Δ15 bp coding (896-910/1482 nt		
AB309	+7			Isolate did not align to reference, may be contaminant. Excluded from analysis.
AB311	+8	G302D (GGC→GAC)		
AB313	+9	Δ9 bp coding (262-270/1482 nt)		
AB315	+10		*rfaP* D187V (GAT→GTT)	
AB298	−1			*argd*[d] Δ134 bp at 2,811,717
AB300	−2			*moaB*[e] Δ1 bp, coding (77/513 nt)
AB302	−3			none
AB304	−4			*yaiX*[f] IS2 insertion (2/416 nt)
AB306	−5			none
AB308	−6			*metE*[g] L19M (CTG→ATG)
AB310	−7			none
AB312	−8			none
AB314	−9			*lplT*[h] (AAACATCATCACCCGGCCTTTGGC)$_{1\to}$ coding (229/1194 nt)
AB316	−10			none

[a]A “Δ” indicates deletion of indicated base pairs or gene. Values in parentheses indicate either single substitutions, or the location of a stop codon, INDEL, or IS-element insertion within coding sequences. Underlined bases indicate single substitutions; “nt” indicates nucleotide.

[b] All *rfa* gene names are synonymous with the corresponding *waa* gene names used in the BW25113 annotation. We report mutations in the *rfa* form for readability (e.g., *waaP* is equivalent to *rfaP*).

[c]*gatR*: pseudogene, repressor for gat operon, interrupted by IS3, split galactitol utilization operon repressor, fragment 2, split galactitol utilization operon repressor, interrupted

[d]*argV*: one of seven arginine tRNAs

[e]*moaB*: inactive molybdopterin adenylyltransferase

[f]*yaiX*: pseudogene, interrupted by IS2A, acetyltransferase homolog, nonfunctional, interrupted by IS2, putative transferase

[g]*metE*: 5-methyltetrahydropteroyltriglutamate- homocysteine S-methyltransferase

[h]*lplT*: lysophospholipid transporter

Table S6. Statistical analysis for phage-mediated trade-offs between colistin resistance and tetracycline resistance. The trade-off is only observed among fluctuation assay mutants ("Fluctuation" model) and not after 10 days of evolution with phage ("Evolution + Phage" model) or in the control populations ("Evolution – Phage" model).

Model	B	R2	p-value	F
Fluctuation	-0.020	0.77	< 0.00001	$F_{1,18} = 58.8$
Evolution + Phage	0.013	0.13	0.31	$F_{1,8} = 1.2$
Evolution – Phage	0	NA	NA	NA

Supplementary Datasets

Dataset S1 (separate file). EOP data on OMP knockouts, corresponding to Fig. 1A.

Dataset S2 (separate file). TolC complementation data, corresponding to Fig. 1B.

Dataset S3 (separate file). Bacterial growth curve data, corresponding to Fig. 1C.

Dataset S4 (separate file). Phage single-step growth curve data, corresponding to Fig. 1D.

Dataset S5 (separate file). EOP data on *rfa* gene knockouts, corresponding to Fig. 2A.

Dataset S6 (separate file). *Rfa* gene complementation data, corresponding to Fig. 2B.

Dataset S7 (separate file). Evolution experiment data, corresponding to Fig. 4.

References

1. Koronakis V., Sharff A., Koronakis E., Luisi B., & Hughes C (2000) Crystal structure of the bacterial membrane protein TolC central to multidrug efflux and protein export. Nature 405:914-919.
